# Genetic control of cellular morphogenesis in Müller glia

**DOI:** 10.1002/glia.23615

**Published:** 2019-03-29

**Authors:** Mark Charlton‐Perkins, Alexandra D. Almeida, Ryan B. MacDonald, William A. Harris

**Affiliations:** ^1^ Department of Physiology, Development and Neuroscience University of Cambridge Cambridge UK; ^2^ Department of Infection, Immunity and Cardiovascular Disease, Medical School and the Bateson Centre University of Sheffield Sheffield UK

**Keywords:** CRISPR, morphogenesis, Müller glia, transcriptome, zebrafish

## Abstract

Cell shape is critical for the proper function of every cell in every tissue in the body. This is especially true for the highly morphologically diverse neural and glia cells of the central nervous system. The molecular processes by which these, or indeed any, cells gain their particular cell‐specific morphology remain largely unexplored. To identify the genes involved in the morphogenesis of the principal glial cell type in the vertebrate retina, the Müller glia (MG), we used genomic and CRISPR based strategies in zebrafish (Danio rerio). We identified 41 genes involved in various aspects of MG cell morphogenesis and revealed a striking concordance between the sequential steps of anatomical feature addition and the expression of cohorts of functionally related genes that regulate these steps. We noted that the many of the genes preferentially expressed in zebrafish MG showed conservation in glia across species suggesting evolutionarily conserved glial developmental pathways.

## 1 INTRODUCTION

The genetic control of postmitotic cell shapes is very poorly understood, especially for the cells making up the central nervous system (CNS), that is, the neurons and glia. These cells assume an immense variety of cell type‐specific morphologies necessary for building their precise connections during development (Kandel, 2013). Glial cells have elaborate morphologies that facilitate their ability to make precise contacts with specific partner neurons, blood vessels, and other glia (Kettenmann & Ransom, 2013). For example, astrocytes, the most abundant glial type in the CNS, emanate numerous fine projections to contact up to two million synapses per cell (Araque, Parpura, Sanzgiri, & Haydon, 1999). These glial projections provide support to their synaptic partners by expressing proteins necessary for energy metabolism, neurotransmitter recycling and ionic homeostasis (Khakh & Sofroniew, 2015). Altered glial morphology is a common pathological feature of neurological disorders and may significantly contribute to neuronal dysfunction and degeneration (Burda & Sofroniew, 2014). Indeed, loss of correct glial morphology and subsequent neuronal support is associated with many psychiatric and neurodegenerative conditions (Bringmann & Reichenbach, 2009; Jadhav, Roesch, & Cepko, 2009; Pfrieger, 2009; Reichenbach & Bringmann, 2013). Despite their importance, it is not well understood how glial cells establish their overall morphology or their precise synaptic contacts during CNS development.

**Figure 1:**
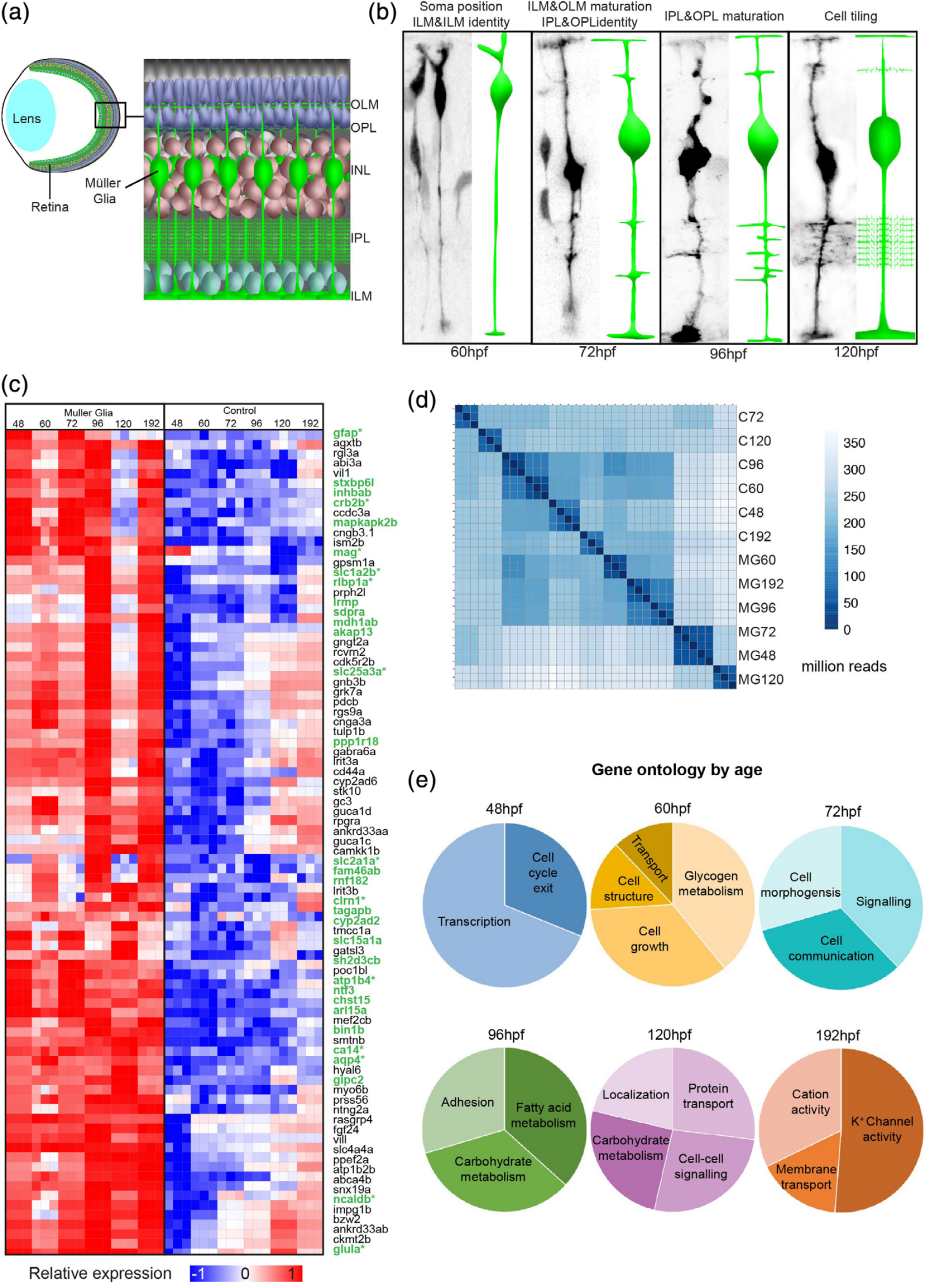
Temporal MG cell morphology and gene expression.(a) Diagrammatic representation of the retina within the eye showing the positioning of MG cells. (b) *Tg*(TP1:Venus) transplanted MG cells showing the time course of MG cell differentiation that gives rise to the distinct MG compartments (OLM: outer limiting membrane; OPL: outer plexiform layer; INL: inner nuclear layer; IPL: inner plexiform layer, ILM: inner limiting membrane). (c) Heatmap (relative expression values by sample—CPM) of top 100 significantly expressed genes in MG (GFAP‐GFP cells) compared to control (GFP negative cells) retinal cells (known glial genes are green and * indicates previous reported expression in MG) (see Supporting Information Table S1 for normalized enrichments). (d) Hierarchical clustering of samples used for RNA‐seq demonstrating consistency between the three replicates used for each time point (MG—GFAP‐GFP sorted cells, C—GFP negative control tissue) (e) Representative gene ontology proportions of MG genes enriched at 48, 60, 72, 96,120, and 196hpf

The radially oriented glial cells in the retina were first described more that 150 years ago (Müller, 1851), and were later named Müller glia (MG) in honor of their discoverer. MG are astonishingly complex and show several elaborate anatomical features that are necessary for precise contact with distinct retinal neurons and membranes. MG are present in all vertebrate retinas and share a conserved set of well‐aligned and layered anatomical features, which were first noted by the great neurohistologist Ramon y Cajal (Cajal, 1892). These features (Figure 1a) include (a) their cell bodies sit in the middle cellular layer of the retina—the inner nuclear layer (INL); (b) their central radial processes span the width of the retina making contact with both the outer to the inner limiting membranes (OLM and ILM); (c) fine branches emerge laterally from these central stalks extending differentially into the two synaptic neuropils, known as the outer and inner plexiform layers (OPL and IPL) (MacDonald, Charlton‐Perkins, & Harris, 2017; Uga and Smelser 1973). Additionally, the MG cells in the mature retina are evenly spaced with their processes arranged in highly ordered mosaics, with little overlap between the cellular domains of neighboring MG cells (Wang et al., 2017; Williams et al., 2010). This network of mature MG morphology facilitates their contact with potentially neuron and synapse in the retina.

The cellular and molecular events underpinning these diverse anatomical features are completely unknown. Yet the conserved, layered, cellular anatomy of MG cells makes them an excellent cell type to investigate the genes involved in cellular morphogenesis (Cajal, 1892; Kolb, Nelson, Ahnelt, & Cuenca, 2001; MacDonald et al., 2015, 2017; Reichenbach & Reichelt, 1986; Wang et al., 2017; Williams et al., 2010). In this study, we combined temporal dissection of the MG developmental process in zebrafish with transcriptomics to identify genes that are significantly enriched in the MG at six key stages of development. Using CRISPR based reverse‐genetic screening of 66 candidates we found that 41 of these genes were implicated in various aspects of MG cell morphogenesis. These studies also reveal that the sequential steps of anatomical feature addition in MG were regulated by successive expression of cohorts of functionally interrelated genes. Furthermore, we identified a conserved Pax2a dependent regulome, previously implicated in vertebrate kidney and invertebrate retinal glia morphogenesis that controls many aspects of zebrafish MG differentiation. Together, our results provide an extensive genetic study that represents the first critical step to furthering our understanding of glial shape formation with potential relevance to general post‐mitotic cell shape acquisition.

## 2 MATERIALS AND METHODS

### 2.1 Animals

Adult zebrafish were maintained and bred at 26.5°C. Embryos were raised at 25–32°C and staged based on (Kimmel, Ballard, Kimmel, Ullmann, & Schilling, 1995) in hours post fertilization (hpf). Embryos were treated with 0.003% phenylthiourea (PTU, Sigma‐ Sigma‐Aldricj, Dorset, UK) from 10hpf to prevent pigmentation. All animal work was approved by the Local Ethical Review Committee at the University of Cambridge and performed according to the protocols of project license PPL 80/2198.

### 2.2 Transgenic lines

Transgenic lines Tg*(atoh7:gap43‐mRFP1)*cu2 (Zolessi, Poggi, Wilkinson, Chien, & Harris, 2006)*,* Tg*(GFAP:GFP) (Bernardos & Raymond,* 2006 *),* Tg*(TP1:Venus‐Pest) (Ninov, Borius, & Stainier,* 2012 *)*.

### 2.3 FACS, RNA‐seq, and bioinformatics

To obtain tissues for FACS and transcriptomic analysis, 20–40 whole eyes of the transgenic zebrafish line *Tg(GFAP:GFP)* were dissected from each developmental time point (48, 60, 72, 96, 120, and 192 hpf) and washed several times to remove debris in L‐15 (Leibovitz's L‐15 Medium). Eyes were then incubated in Trypsin‐EDTA 0.25% (Sigma) at 37°C for 15 min, washed several times and dissociated using FBS coated pipette tips in Calcium‐free medium (116.6 mM NaCl, 0.67 mM KCl, 4.62 mM Tris, 0.4 mM EDTA). Single cell suspensions were sorted on a Beckman Coulter MoFlo to capture Müller glia (GFP) and control (C) retinal tissue (non‐GFP). Cells were sorted into lysis buffer, and RNA was immediately extracted using the RNeasy mini kit (Qiagen, Manchester, UK). RNA concentration and qualities were assessed on an Agilent Bioanalyzer, and RNA amplification and cDNA synthesis were performed with the Ovation RNA Amplification System V2 (NuGEN, Leek, Netherlands) using the manufacturer's protocol. Nextera library preparations were performed using the Nextera DNA library kit according to the manufacturer's directions and sent to the Sanger Centre for sequencing.

Sequence files (GEO accession GSE120275) were paired, trimmed and aligned using Hisat2 to the zebrafish genome (version: Zv9) and RNA‐seq bioinformatic and statistical analysis was performed in R using the Bioconductor, FeatureCounts, Rsubread, limma, DESeq2, DEFormats, pheatmap, ggplots, org.Dr.er.db, and EdgeR packages. For enrichment analysis, trimmed‐means‐of‐m (TMM) analysis was combined with Benjamin‐Hochberg correction to correct false discovery rate (FDR). Cross‐species gene conversions were performed using Ensembl (Biomart, Ensembl resource https://www.ensembl.org/) by using zebrafish (GRCz11) or fly (BDGP5) gene symbols as the input filter, and Ensembl gene ortholog (mouse) readout attributes. Overlaps were between separate datasets were then quantified using Venny (2.1.0). Statistical significance of gene overlaps was calculated using Fisher's exact tests and corrected to discount multiple testing errors using the strict Bonferroni method. Gene Ontology analysis and statistics were performed using Gene Ontology Consortium (Ashburner et al., 2000; The Gene Ontology Consortium, 2017).

### 2.4 Embryo manipulations

For blastomere transplantations, high‐ to oblong‐stage embryos were dechorionated by pronase digestion (Sigma), placed in agarose molds, and between 5 and 30 blastomeres were transferred between Tg*(TP1:Venus)* embryos and wild type embryos using a glass capillary connected to a 2 mL syringe. Embryos were grown on dishes coated with 1% agarose in 0.04% PTU overnight until imaged by confocal microscopy.

### 2.5 sgRNA design and reverse genetic screen

The sgRNA design and strategy are primarily based on the methods from Shah and colleagues (Shah, Davey, Whitebirch, Miller, & Moens, 2016). Briefly, each guide RNA was designed using the ChopChop design tool (Labun, Montague, Gagnon, Thyme, & Valen, 2016) at chopchop.cbu.uib.no/index.php. For each gene, the two gRNAs with minimal predicted off‐target sites were selected and co‐injected. In the first screen, we picked the two targets with the highest overall rankings in the first exon, while in the second and third screens we used the highest‐ranking targets for the first and last exon of each gene. Template DNA was synthesized by in vitro transcription of a two oligo PCR method. For this, an oligo scaffold containing the RNA loop structure 5′[gatccgcaccgactcggtgccactttttcaagttgataacggactagccttattttaacttgctatttctagctctaaaac]3′ required for Cas9 was synthesized and used for the syntheses of all gRNAs (Extended data Table 2). Next, a unique oligo containing the T7 promoter, the 20 nucleotides gRNA, and 20 bases of homology to the scaffold oligo was synthesized. PCR amplification of these annealed oligos sequence was created using Phusion master mix (New England Biolabs, M0531 L) with 10uM scaffold and gRNA for 40 cycles in a thermal cycler. This PCR product was purified (PCR purification kit; Qiagen) and used as a template for the in vitro transcription reaction (T7 megascript; Ambion). RNA was purified on columns (Zymo Research, D4014) and injected using 100 pg of each gRNA (200 ng total) with 1200 pg of Cas9 encoding mRNA.

### 2.6 Immunostaining and microscopy

Imaging of CRISPR injected embryos for each screen was carried out on fixed embryos at 120hpf (4% paraformaldehyde). Embryos were mounted in 1% low melting point agarose and positioned to allow for imaging of the retina in situ.

For immunohistochemistry CRISPR injected and mutant embryos were fixed at 120hpf in 4% paraformaldehyde overnight at 4°C, washed in PBS and then stored in MeOH at −20°C. Samples were re‐hydrated in a MeOH:PBS series (3:1, 1:1, 1:3) followed by three PBST (PBS + 0.05% Triton‐X100) washes. Rehydrated whole embryos were incubated in GFP‐Booster Atto488 (1:500, Chormotek) for 2 hr at RT and were then mounted on slides with a coverslip bridge (to prevent crushing the tissue) in Prolong Diamond (Invitrogen) and allowed to cure at room temperature overnight before imaging. For Pax2 staining samples were incubated in Rabbit anti‐Pax2 (1:200; previously Covance catalog# PRB‐276P) and goat anti‐rabbit conjugated Alexa Fluor 555 1:500 (Invitrogen) at 4°C overnight and mounted as above.

Laser scanning confocal imaging was performed using an Olympus FV1000 microscope with a ×60 oil objective (1.35 NA). For live imaging, optical sections at 0.5–1 μm separation were taken to cover the region of the retina containing the cells of interest (between 10 and 30 μm) every 15 min over 12 hr. For CRISPR screening, 0.5um optical sections of transverse sections near the middle of the retina on whole embryos, which were orientated so that the outer surface of the eye was closest to the coverslip, as described previously (Das, Payer, Cayouette, & Harris, 2003). Confocal data were analyzed and processed using Volocity (PerkinElmer) and ImageJ/FIJI (NIH).

### 2.7 Phenotype analysis

For phenotype analysis, injections of gRNAs were made in three replicate experiments. Control animals were injected with guide RNAs to *Slc24a5,* which resulted in normal MG morphology and loss of animal pigment confirming that our CRISPR strategy was effective (Supporting Information Figure S4a). Similar to previous CRISPR screening techniques, control and experimental injections produced between ~30 and ~55% embryonic lethality with phenotypes observable in around 95% of the surviving animals (Supporting Information Figure S4f; Shah et al., 2016; Wu et al., 2018). We also generated F1 mutant lines for several CRISPR mutants (*pax2a*, *nphs1*, *kirrela*, *Itga5*, *Itga6*, *wt1b*, *cadm1b,* and *cadm4*) and confirmed that CRISPR mutation was highly specific (by DNA sequencing) and 100% penetrant (by phenotype similarity) (Supporting Information Figure S4e; Shah et al., 2016). Finally, *pax2a* CRISPR mutation was verified by the fact that Pax2 immunostaining at 72hpf shows positive nuclei in control animals but mostly absent in F0 CRISPR injected fish and completely absent in F1 *pax2a* mutants (Supporting Information Figure S4b–d).

Phenotype counts were done on between 45 and 56 injected animals for each mutant (Supporting Information Table S4) using un‐flattened (un‐processed) z‐stack transverse images of retinas in situ in whole embryos. Each sample's MG features were scored throughout the tissue using the following criteria to make a decision on the presence or absence of particular defects: Soma position: Abnormal MG soma position basally or apically, adjacent to the IPL, or completely displaced from the INL; OLM: Large breaks or the complete absence of the OLM; ILM: Large breaks or the complete absence of the ILM; IPL: Altered thickness, failure to elaborate, or abnormal elaboration of the IPL; OPL: Failure or abnormal elaboration of the OPL; Tiling: Significant spacing disruptions (large gaps or multiple overlaps) of MG cell bodies, and/or MG projections including those in the IPL and OPL. Mutant phenotype counts were subjected to Fisher's exact tests to quantify significance with Bonferroni correction to eliminate multiple testing errors (121 for Screen 1, 174 for Screen 2, 120 for Screen 3).

## 3 RESULTS

### 3.1 The anatomical development of MG can be broken into sequential steps of feature addition

It is known that Notch signaling is essential for MG cell specification (Dorsky, Rapaport, & Harris, 1995; Ohnuma, Philpott, Wang, Holt, & Harris, 1999; Vetter & Moore, 2001) and in the Tg(*TP1:Venus)* transgenic line (Ninov et al., 2012), the Notch‐responsive element TP1 drives expression of the fluorescent protein, Venus, allowing MG cells to be followed from the time of their initial specification in zebrafish at ~60 hpf (MacDonald et al., 2015). By transplanting blastomeres from the transgenic zebrafish line into wild‐type hosts, we were able to visualize the sequential steps of MG morphogenesis in zebrafish in vivo (Figure 1b). At 60hpf, the MG cell bodies begin to migrate basally to their stereotypic position in the middle of the INL of the retina (MacDonald et al., 2015). By 72hpf, MG begin to expand their apical processes and basal endfeet to confluently fill the surfaces of the OLM and ILM, respectively. At this time, they also begin to extend dynamic filopodia from their central stalks, which identify the OLP and the apical and basal limits of the IPL. By 96hpf MG in zebrafish elaborate fine processes with the plexiform layers (Williams et al., 2010). The last step that we investigated in this process is that MG cells space themselves out across the retina such that they are evenly distributed across the retina with little to no gaps or overlaps (MacDonald et al., 2015; Williams et al., 2010). Homotypic repulsive cell interactions are thought to account for this (Bushong, Martone, Jones, & Ellisman, 2002; Williams et al., 2010), as focal ablation of MG cells results in nearby MG cells extending processes to fill in the spaces previously occupied by the ablated MG cell (Williams et al., 2010). Thus, by the time robust vision commences in zebrafish, about 120hpf (Biehlmaier, Neuhauss, & Kohler, 2003), these cells have gained the full set of the conserved cell‐specific anatomical characteristics that Cajal originally identified.

### 3.2 Transcriptomic analysis of key stages in MG morphogenesis reveals gene ontology differences

To search for genes involved in MG cell morphogenesis, we FACS‐sorted MG at specific times (48, 60, 72, 96, 120, and 192hpf) that span the morphogenetic process outlined above, and identified genes expressed preferentially at each of these time points (Figure 1c). Hierarchical clustering and principal component analysis of these data reveal that minimal differences in three experimental replicates of these individual time‐points (Figure 1d). However, significant differential gene expression is notable throughout MG cell differentiation (Supporting Information Table S1). Importantly, clustering all time‐points by trimmed means of M (TMM) differential gene enrichment, shows that several of the top 100 genes have previously been associated directly with MG cells or other glia (Figures 1c, Supporting Information Table S2) (Dahlin, Royall, Hohmann, & Wang, 2009; Eisenfeld, Bunt‐Milam, & Sarthy, 1984; Jo et al., 2015; Lehre & Danbolt, 1998; Lehre, Davanger, & Danbolt, 1997; Riepe & Norenburg, 1977; Saari et al., 2001; White & Neal, 1976; Zong et al., 2010). Overrepresentation of the gene ontology terms revealed several terms that are frequently associated with differentiation including; transcription, cell cycle exit, cell structure, adhesion, metabolism, growth signaling, membrane transport, and cation activity.

The Gene Ontology (GO) terms for each developmental stage revealed dynamic changes in the biological and molecular functions of differentially expressed genes throughout MG development (Figure 1e, Supporting Information Table S3). Focusing, for example, on the gene ontologies that make intuitive sense, we find at 48hpf cell cycle genes were over‐represented, at 60hpf it was genes involved in cell growth, at 72hpf cell signaling, at 96hpf adhesion, and at 120hpf and 196hpf physiological function. Other gene ontologies involved in metabolism and transport also changed expression over this time course.

### 3.3 CRISPR analysis of phenotypes during MG development

To determine if the temporal expression of these genes is important for discrete stages of MG morphogenesis, we used hierarchical clustering and TMM differential expression analysis to identify genes that first became enriched at specific time points and remained enriched until 192hpf (i.e., 48–192, 60–192, 72–192, and 96–192hpf) (Supporting Information Table S4). We then limited our attention to those genes that code for proteins that seemed likely candidates to have an impact on cell morphogenesis (Supporting Information Table S4), and knocked out these genes by injecting Cas9 mRNA and candidate‐specific gRNAs (Shah et al., 2016; Wu et al., 2018). The background was always the Tg*(GFAP:GFP)* transgenic line (Bernardos & Raymond, 2006) so that we could assay morphological defects specifically in MG cells in F0 embryos at 120hpf (see methods for CRISPR screening validation). The CRISPRed fish all continued to express the GFAP : GFP transgene in MG cells, suggesting initial glial specification is unaffected in any of the F0 CRISPR mutants. Yet many of the F0 CRISPR mutants produced clear defects in MG cell anatomy. Analyzing 45 to 60 injected animals per CRISPR, we scored the frequency that obvious phenotypes were observed in accordance with the particular criteria for each morphological feature (see Methods, Supporting Information Table S5).

**Figure 2:**
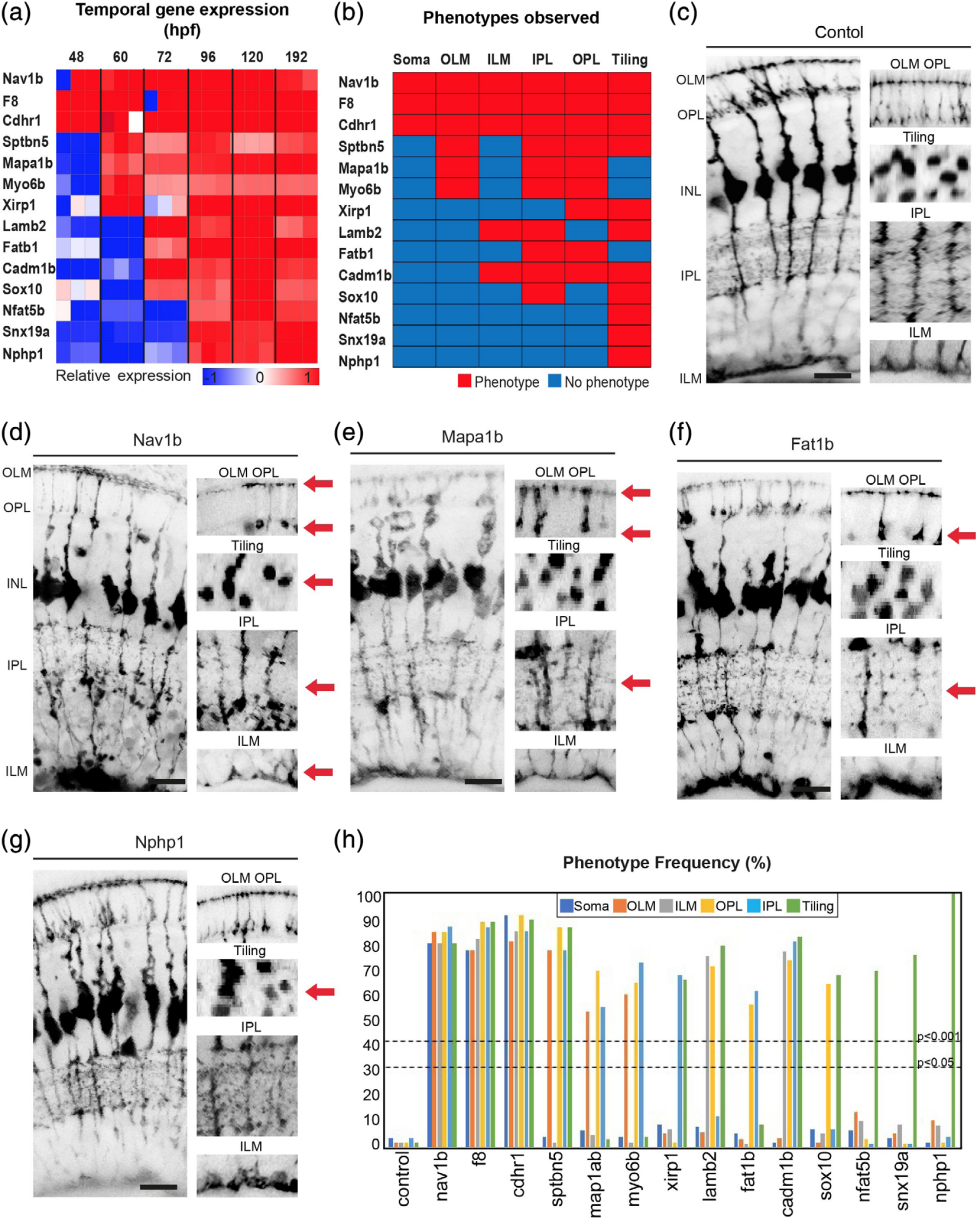
Temporal gene expression dictates MG cell morphologies. (a) Heatmap to show the relative gene expression for genes tested. (b) Summary of phenotypes observed for genes enriched across windows of MG cell differentiation. Red—phenotype, blue—no‐phenotype. (c) *slc24a5* CRISPR injected control animals have normal MG cell morphology that extends from the apical to the basal surfaces, forming the ILM (inner limiting membrane) and OLM (outer limiting membrane) on either side. MG cells are also regularly tilled across in the eye with their cell bodies mostly restricted to the middle of the INL (inner nuclear layer) and are highly branched within the IPL (inner plexiform layer) and OPL (outer plexiform layer). (d) *nav1b* CRISPR injected animals have defects in apico‐basal cell body position in the INL (inner nuclear layer), OLM (outer limiting membrane), OPL (outer plexiform layer), tiling, IPL (inner plexiform layer), and ILM (inner limiting membrane). (e) *mapab1* CRISPR injected animals have defects in OLM, OPL, and IPL. (f) *fat1b* CRISPR injected animals have defects in OPL and IPL defects. (g) *nphp1* CRISPR injected animals have defects in MG cell tiling. (h) Frequency (%) of phenotypes observed in each MG compartment in F0 CRISPR screen 1. Scale bars = 8μm

***48–192hpf***. Genes enriched from 48–192hpf with mutant phenotypes include *nav1b*, *f8*, and *cdhr1*. CRISPRed F0s for these genes produced irregularly shaped MG cells with significant defects in many of the cells conserved morphological features (Figure 2a–d; Supporting Information Figure S1b,c).

***60–192hpf***. Genes enriched from 60–192hpf with mutant phenotypes include *sptbn5*, *myo6b*, *xirp1*, and *map1ap* (Figure 2a). Interestingly none of them showed significant apico‐basal soma positioning defects though they had several other defects (Figure 2b,e; Supporting Information Figure S1d–h).

***72–192hpf***. Genes enriched from 72–192hpf with significant mutant phenotypes include *lamb2*, *fat1b*, *cadm1b*, and *sox10* (Figure 2a). These displayed defects in still fewer and later aspects of MG cell morphogenesis (Figure 2b,f; Supporting Information Figure S1g–i).

***96–192hpf***. Genes enriched from 96–192hpf with significant mutant phenotypes include *nfat5c*, *snx19a*, and *nphp1* (Figure 2a). All these only showed defects in MG cell tiling most notably in the spacing of the soma and/or overlapping inner and outer plexiform layers. (Figure 2b,g; Supporting Information Figure S1j,k).

The frequency of defects in each MG compartment was independently quantified to determine each overall mutant phenotype and 14 of the 21 candidates tested in the initial screen had significant defects (Figure 2h, Supporting Information Table S5). These results indicate a gradation of phenotypes such that genes enriched at early stages are involved in multiple defects in MG cell morphogenesis whereas those enriched later genes have roles that are restricted in late developing features of MG.

**Figure 3:**
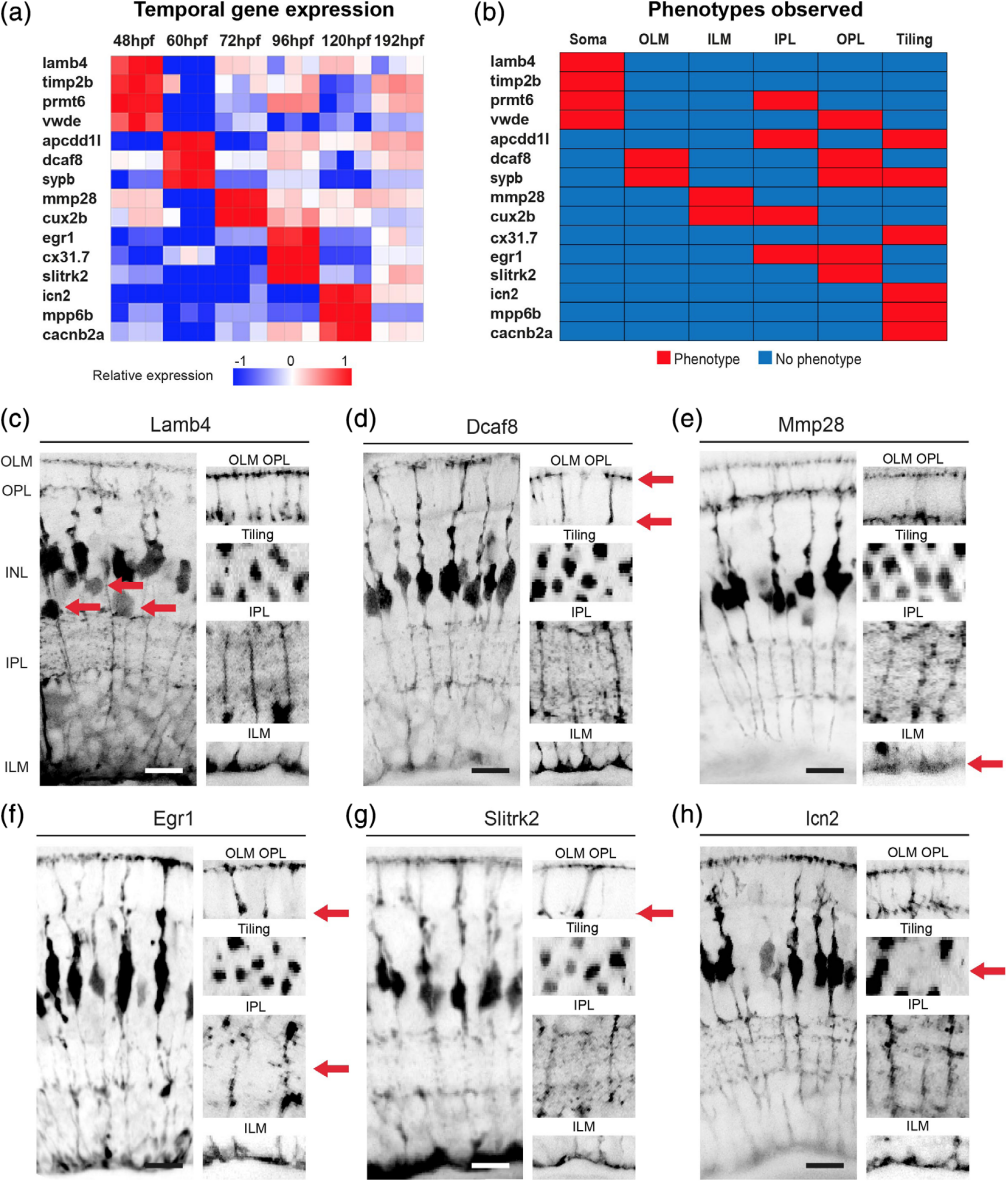
Discrete gene expression regulates MG cell compartment morphology. (a) Heatmap to show the relative gene expression for genes tested. These were all screen in F0 CRISPR injected mutants. (b) Summary of phenotypes observed for genes enriched across windows of MG differentiation. Red—phenotype, blue—no‐phenotype. (c) *lamb4* mutants have defects in the apico‐basal distribution of MG cell bodies only. (d) *dcaf8* mutants have defects in the OLM and OPL. (e) *mmp28* CRISPR injected mutants have defects in the ILM only. (f) *egr1* mutants have defects in the IPL and OPL. (g) *slitkr2* mutants have defects in the OPL layer only. (h) *icn2* mutants have tiling defects only. 1. Frequency (%) of phenotypes observed in each MG compartment in F0 CRISPR screen 2. Scale bars = 8 μm

We next asked whether genes enriched during more narrowly restricted time windows would have more refined morphogenetic defects (Figure 3a, Supporting Information Table S4, Supporting Information Figure S2k). For this, we used hierarchical clustering and TMM analysis to identify the genes that were only enriched at a single individual time point in MG.

***48hpf***. Two genes enriched at 48hpf, *lamb4,* and *timp2b* showed significant defects in apical‐basal soma positions (Figure 3b,c; Supporting Information Figure S2B). Apical‐basal soma defects were also seen in mutants of two other genes that were transiently enriched at 48hpf: *prtm6a* and *vwde* (Figure 3b; Supporting Information Figure S2c,d). However, *prmt6* mutants also show defects in ILM formation, and *vwde* mutants also show defects in OPL formation (Figure 3b, Supporting Information Figure S2c,d; Supporting Information Table S4).

***60hpf***. Genes that were overrepresented at 60hpf with mutant phenotypes showed defects in later stages of morphogenesis (Figure 3b). *dcaf8* mutants have OPL and OLM defects (Figure 3d; Supporting Information Table S4), *apcdd1l* mutants have defects in IPL and MG tiling (Supporting Information Figure S2e; Supporting Information Table S4), and *sypb* mutants show defects in OPL, OLM, and tiling, (Supporting Information Figure S2f; Supporting Information Table S4).

***72hpf***. Mutants in genes enriched at 72hpf preferentially affected later steps of morphogenesis: *mmp28* mutants affected the ILM (Figure 3e; Supporting Information Table S4), and *cux2b* mutants affected the ILM and IPL in (Figure 3b; Supporting Information Figure S2g).

***96hpf***. Similarly, mutants in genes enriched at 96hpf, *cx31.7*, *egr1*, and *slitrk2,* showed subtle defects in the IPL and OPL, and tiling (Figure 3a,f, and g; Supporting Information Figure S2h; Supporting Information Table S4).

***120hpf***. Finally, mutant in genes preferentially expressed at 120hpf, including *icn2*, *mpp6b*, and *cacnb2a* (Figure 3a) produce nothing more than tiling defects, (Figure 3h; Supporting Information Figure S2g; Supporting Information Table S4; Supporting Information Table S4).

The frequency of defects in each MG compartment was again quantified for each mutant with 15 of the 29 candidates in this second screen showing statically significant phenotypes (Figure 3i, Supporting Information Table S5). Together, these data reveal a striking correlation with the type of phenotype seen, the temporal expression of specific genes, and the developmental time course of the addition of specific anatomical features. This suggests that particular features of cellular anatomy invoke the transcription of specific genetic repertoires that work at particular periods of development.

### 3.4 Conserved regulators of glial morphogenesis

**Figure 4:**
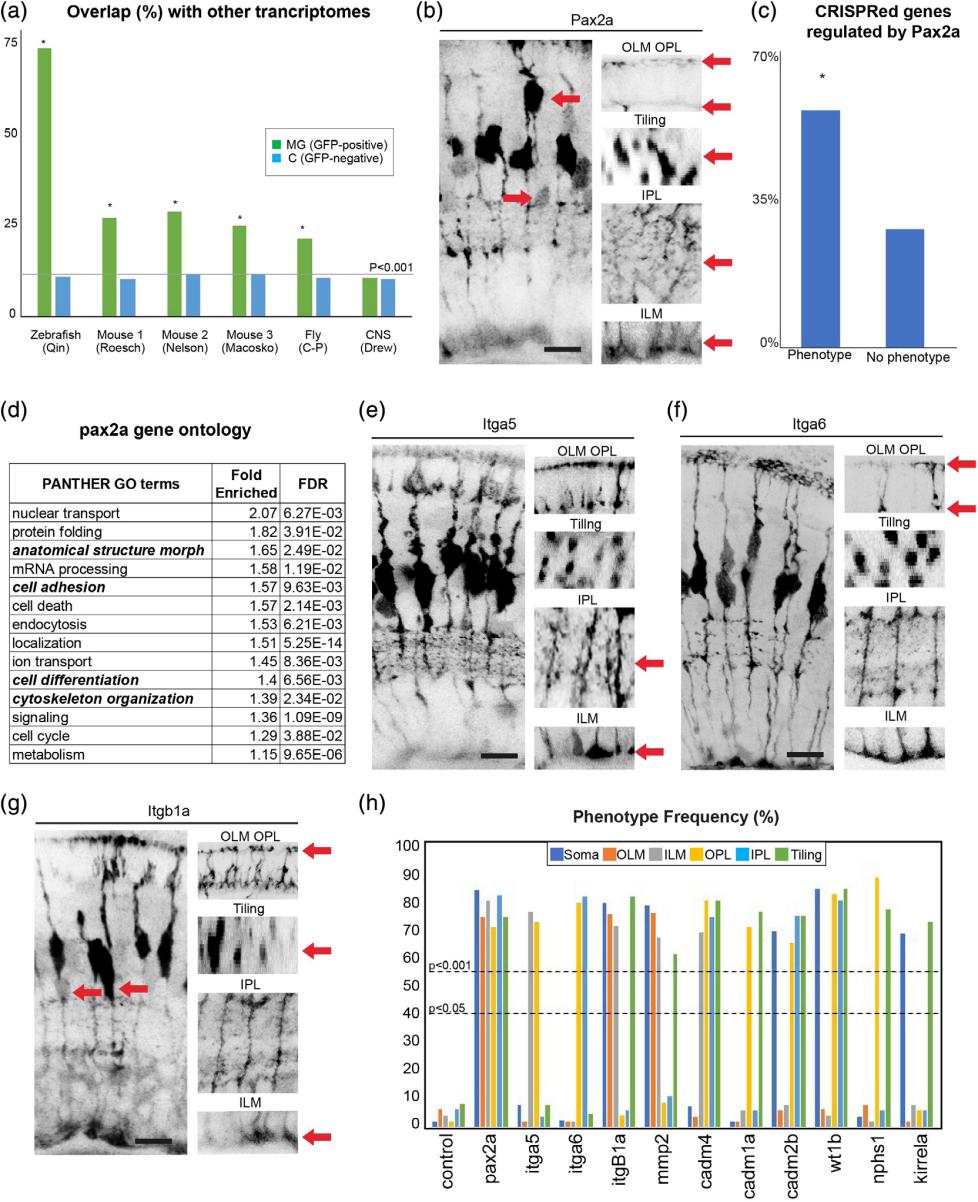
A set of highly conserved genes that affect MG cell morphology. (a) Overlap of zebrafish MG enriched genes with previously reported MG transcriptomes from zebrafish, mouse, and fly (Macosko et al., 2015; Nelson et al., 2011; Qin, Barthel, & Raymond, 2009; Roesch et al., 2008; Sifuentes, Kim, Swaroop, & Raymond, 2016). MG—genes enriched in GFAP‐positive cells; C—Genes enriched in non‐GFP positive cells; * indicates significance (Bonferroni adjusted *p*‐value <0.001) by Fisher's exact test. (b) *pax2a* CRISPR injected animals have highly disorganized retinas with breaks in the OLM and ILM, abnormal tiling and apico‐basal distribution of the cell bodies, as well as much less branching in the IPL and OPL (see Supporting Information Table S4 for details). (c) Percentages of genes used in this study that either had or did not have a phenotype. * indicates significance by Fisher's exact test. (d) GO terms for the top 500 genes significantly (adjusted *p* < 0.05) up or down‐regulated *pax2a* mutants. (e) *itga5* CRISPR injected animals have defects on the basal side of MG specifically in the ILM and IPL. (f) Itag6 CRISPR injected animals have defects on the apical side of the cell in the OLM and OPL. (g) F0 *itb1a* CRISPR injected animals have defects in cell body tiling and apico‐basal position, as well as in OLM and ILM. (h) Frequency (%) of phenotypes observed in each MG compartment in F0 CRISPR screen 3. Scale bars = 8μm

We noted that a large fraction of the genes identified proved to be essential for MG cell morphogenesis, as well as many that we did not test using CRISPR analysis, were highly conserved regarding their expression in glial cells across species (Figure 4a; Supporting Information Figure S3a; Supporting Information Table S4). For instance, from our transcriptome, control and MG genes have similar levels of overlap with zebrafish whole CNS genes (Figure 4a; Drew et al., 2008). However, in cross‐species comparisons, we find a highly significant overlap of the MG orthologs with other glial datasets from zebrafish, mice, the fly retina, while the control (mixed retinal population) tissue overlap has no significant difference in the overlap between MG and the whole CNS (Figure 4a; Roesch et al., 2008; Qin et al., 2009; Nelson et al., 2011; Macosko et al., 2015; Sifuentes et al., 2016; Charlton‐Perkins, Sendler, Buschbeck, & Cook, 2017). Some of these highly conserved genes are known to be involved in glial differentiation. For example, *Pax2a* (from the Pax2/5/8 family) and the integrins (Itga5, Itga6, and Itgb1a) are expressed in many glial cells, and mutants in all of these genes also resulted in defects in many aspects MG cell morphology (Figure 4b; Supporting Information Figure S3b–e; Supporting Information Table S4; Supporting Information Figure S3h; Charlton‐Perkins et al., 2011, 2017; Putaala, Soininen, Kilpeläinen, Wartiovaara, & Tryggvason, 2001; Quaggin, 2002; Ambu et al., 2015; Dzyubenko, Gottschling, & Faissner, 2016). Remarkably, analysis of the transcriptome of MG cells in *pax2a* mutants shows that 60% of the genes that affect MG cell morphogenesis in our study have significant changes of expression (Figure 4c; Supporting Information Table 4). In addition, the differentially expressed genes in *pax2a* mutant MG cells show a significant enrichment of GO terms related to cell morphology, adhesion, and differentiation (Figure 4d; Supporting Information Table S5). These results suggest that Pax2 is a key regulator of MG morphogenesis. We also found several other families of conserved glial genes involved in various aspects of MG morphogenesis: for instance, *nephrins* (Supporting Information Figure S3c,d), and the Cadm family of cell adhesion molecules (Supporting Information Figure S3g–i). We quantified MG compartment defect frequency for all of these and found 11 with statistically significant phenotypes (Figure 4h, Supporting Information Table S5). Together, these results suggest that there are many conserved molecular genetic principles of glial cell morphogenesis.

## 4 DISCUSSION

The post‐mitotic temporal addition of layered morphological features in MG offers a unique opportunity to genetically dissect the poorly understood process of cell morphogenesis in zebrafish where development is rapid and the embryos is transparent. Indeed, in mice, MG differentiation begins at P0 and lasts beyond P9 and many of the details of this process have not yet been studied (Nelson et al., 2011). However, similarly to the zebrafish MG differentiation, it has been noted that the ILM/OLM formation occurs prior to P7, IPL elaboration begins around P7, and OPL elaboration begins around P8 (Wang et al., 2017). Using our approach combining cell sorting, transcriptomics, and CRISPRs, we identified many key players in fish MG differentiation, and linked them to specific temporal windows of action, during which morphological features are added. We therefore suspect that future studies in mammal MG differentiation will find many orthologous genes with similar functions.

The temporal transcriptome and bioinformatic analysis here are the first in‐depth dataset of cell morphogenesis. Sequencing technologies have improved dramatically in the past few years such that combined with bioinformatic normalizations and false discovery rate (FDR) correction there is little question about the timing and enrichment of MG genes found here (Salk, Schmitt, & Loeb, 2018). However, there is certainly information that we cannot get from transcriptomics that needs to be included in future morphogenesis studies to improve our understanding of glial morphogenesis. One example of this is that of the integrin pathway which is already known to regulate the placement and morphology of glia across the nervous system (Dzyubenko et al., 2016). The integrin complex is made up of alpha and beta subunits that serve as signaling and adhesive attachments between the cell and the extracellular matrix (Giancotti & Ruoslahti, 1999). Here we find three subunits, two alpha (Itga1a, Itga1b) and one (Itgb1a) subunit (Figure 4e,f) that together have phenotypes in every MG compartment. However, the alpha‐subunits produce phenotypes in more distinct MG compartments than the beta‐subunit suggesting that beta‐subunits are more broadly expressed across the cell membrane. This fits well with the previous knowledge that integrin complexes are made of different subunits depending on the part (apical or basal) of the cell surface they reside (Lowell & Mayadas, 2012; Salk et al., 2018). Thus, future studies using focusing of these and other cell adhesion families identified in this study should provide a more detailed understanding of their roles in glial cell morphology.

CRISPR/Cas9 technology has added an invaluable tool that is quickly replacing traditional morpholino analysis in zebrafish and other model organisms. If done carefully, it has very few off‐target effects, and its accuracy is currently undisputed (Anon, 2018). For these reasons, it is unlikely that we have many off‐target effects in this study. This possibility seems even less likely considering the high incidence and the morphological specificities of the MG phenotypes seen. Our screening also reliably produced the same F0 morphological defects in the MG population, and we have confirmed these in some several F1 generations (Supporting Information Figure S4e). It should be noted that the F0 CRISPR mutants are by nature mosaic, and the specific morphologies of the MG defects seen in our F0 screens need to be examined in fully mutant lines. Such lines can also be used in studies to test the cell‐autonomous versus non‐autonomous aspects of these phenotypes by transplanting blastomeres from mutant lines into normal hosts and vice‐versa. Indeed, none of the particular phenotypes found in this study was quantitatively or mechanistically investigated further, as this did not seem reasonable in an F0 screen. More detailed quantitative and mechanistic investigations into these phenotypes will be the subject of future studies.

The high level of genetic conservation of glia in the animal kingdom suggests there may be basic principles of glial biology (Charlton‐Perkins et al., 2017), and the finding here that several conserved genes are involved during particular temporal windows of MG morphogenesis, suggest that there may also be conserved programs of differentiation. A recent study has implicated some proteins found here in mouse MG morphology and tiling including Rbx2, Dab1, and SOCS7 (Fairchild et al., 2018). Another excellent example of this conserved function is Pax2, which in the eye is primarily known for its function in optic stork formation, and whose expression has been noted in mature MG of chick but not guinea pigs or dogs (Boije, Ring, López‐Gallardo, Prada, & Hallböök, 2010; Stanke, Moose, El‐Hodiri, & Fischer, 2010). Pax2 and Wt1 are also crucial for cellular patterning through their regulation of the Nephrins in the brain, kidney, and fly retinal glia (Ambu et al., 2015; Bao & Cagan, 2005; Cagan, 2003; Charlton‐Perkins et al., 2011, 2017; Flores, Daga, Kalhor, & Banerjee, 1998; Fu & Noll, 1997; Putaala et al., 2001; Quaggin, 2002; Wagner et al., 2002). Our transcriptomes indicate that many Nephrins are also temporally enriched in zebrafish MG, they affect glial morphology, and their expression is Pax2a dependent (Supporting Information Table S4). Taken together, our data suggest that Pax2, Wt1, and the Nephrins are part of a conserved regulome that controls cell shape and patterning in multiple biological contexts. It would, therefore, be fascinating to understand the genetic relationship between Nphp1 and other “kidney related” genes in future more in‐depth compound genetic studies of all the above genes.

Perhaps the most remarkable finding of this study is the enrichment of genes during narrow windows that regulate the differentiation of discrete MG compartments that develop in those time windows, although in retrospect it seems obvious that pathways that come on late in MG development could not possibly affect early stages of MG development. Single‐cell transcriptomic studies have shown a level of background variability between individual cells (Tasic, 2018). However, in the context of this study, we find that the MG follow rather precise changes in gene expression that temporally correlate with MG compartment differentiation. Our analyses suggest that successive steps of cell morphogenesis are due ti the timing of the expression of cohorts of conserved interrelated genes that have roles in generating the particular anatomical features of these cells and that a sequence of genetic regulomes govern stepwise cellular morphogenesis in this system. We hope that this work, which suggests the development MG can be approached stage‐by‐stage and feature‐by‐feature, each stage and feature with its own development genetic programs, will provide a foundation for future mechanistic studies of cellular morphogenesis.

## CONFLICT OF INTEREST

The authors declare no conflict of interests.

## Supporting information


**Supplemental figure 1:** Phenotypes of gene mutants enriched over windows of MG cell differentiation. (a) *slc45a5* controls have no observable MG phenotype. (b) *f8* mutants have defects in cell body position, OLM, ILM, IPL, OPL, and tiling. (c) *cdhr1* mutants have defects in cell body position, OLM, ILM, IPL, OPL, and tiling. (d) *sptbn* mutants have defects in OLM, IPL, OPL, and tiling. (e) *mapa1b* mutants have defects in OLM, IPL, and OPL. (f) *xirp1* mutants have defects in OPL and tiling. (g) *lamb2* mutants have defects in ILM, IPL, and tiling. (h) Cadm1b mutants have defects in ILM, IPL, OPL, and tiling. (i) *sox10* mutants have defects in IPL and tiling. (j) *nfat5* mutants have tiling defects. (k) *snx19a* mutants have tiling defects. (l) Percentages of individual phenotypes observed in all animals from this screen. Dashed lines represent levels of significance from Fisher's exact test after Boniforni multiple test correction (bottom = *p* < 0.05, top = *p* < 0.001). Scale bars = 8μm.

**Supplemental figure 2:** Phenotypes of gene mutants that are enriched at specific times of MG differentiation. (a) *slc45a5* controls have no observable MG phenotype. (b) *timp2b* mutants have defects in cell body position. (c) *prmt6* mutants have defects in cell body position and ILM. (d) *vwde* mutants have defects in cell body position, OLM, ILM, IPL, OPL, and tiling. (e) *apcdd1l* mutants have defects in IPL and OPL. (f) *sypb* mutants have defects in OLM, OPL, and tiling. G) Cux2b mutants have defects in ILM and IPL. (h) *cx31.7* mutants have defects in tiling. (i) *Mpp6b* mutants have defects in tiling. (j) *cacnb2a* mutants have defects in tiling. (h) Percentages of individual phenotypes observed in all animals from this screen. Dashed lines represent levels of significance from Fisher's exact test after Boniforni multiple test correction (bottom = *p* < 0.05, top = *p* < 0.001). Scale bars = 8μm.

**Supplemental figure 3:** Phenotypes of conserved highly conserved MG cell genes. (a) Schematic representation of how highly conserved genes we bioinformatically identified. (b) *slc45a5* controls have no observable MG phenotype. (b) *timp2b* mutants have defects in cell body position. (c) *nphs1* mutants have defects in ILM, IPL, and tiling. (d) *kirrela* mutants have defects in cell body position and tiling. (e) *wt1* mutants have defects in cell body position, IPL, OPL, and tiling. (f) *mmp2* mutants have defects in OLM, ILM, and tiling. (g) *cadm4* mutants have defects in OPL, IPL, ILM, and tiling. (h) Cadm1a mutants have defects in IPL and tiling. (i) *cadm2b* mutants have defects in a cell body positing, IPL OPL and tiling. (j–l) Percentages of individual phenotypes observed in all animals from this screen. Dashed lines represent levels of significance from Fisher's exact test after Boniforni multiple test correction (bottom = *p* < 0.05, top = *p* < 0.001). Scale bars = 8μm.

**Supplemental figure 4** CRISPR injection validation. (a) Cas9 only injected F0 fish have normal pigmentation at 120hpf while those injected with Cas9 and the *slc45a5* guide RNAs are mostly devoid of pigment. (b) In control animals (GFAP:GFP) Pax2 is expressed in all MG by 120hpf. (c) F0 *pax2a* CRISPR injected animals lack Pax2 expression in most, but not all MG. (d) F1 *pax2a* CRISPR injected animals Pax2 is absent from all MG. (e) F1 CRISPR mutants with confirmed mutations have notably similar defects to those identified in F0 screen 3. (f) Percentages of animals with lethality and phenotypes after injections. Scale bars = 8μm.

**Supplemental Table 1** TMM fold‐change analysis of each MG cell developmental stage.

**Supplemental Table 2** Log counts per million for all samples

**Supplemental Table 3** Gene ontologies of each stage of MG cell differentiation.

**Supplemental Table 4** Summary of all genes used with gRNAs and phenotypes.

**Supplemental Table 5** TMM gene enrichments of Pax2a mutant MG cells
