# Supplementary figures and images for: Genetic control of cellular morphogenesis in Müller glia
Source: Glia. 2019 Mar 29;67(7):1401–11. doi: 10.1002/glia.23615 (PMC6563441; doi:10.1002/glia.23615)

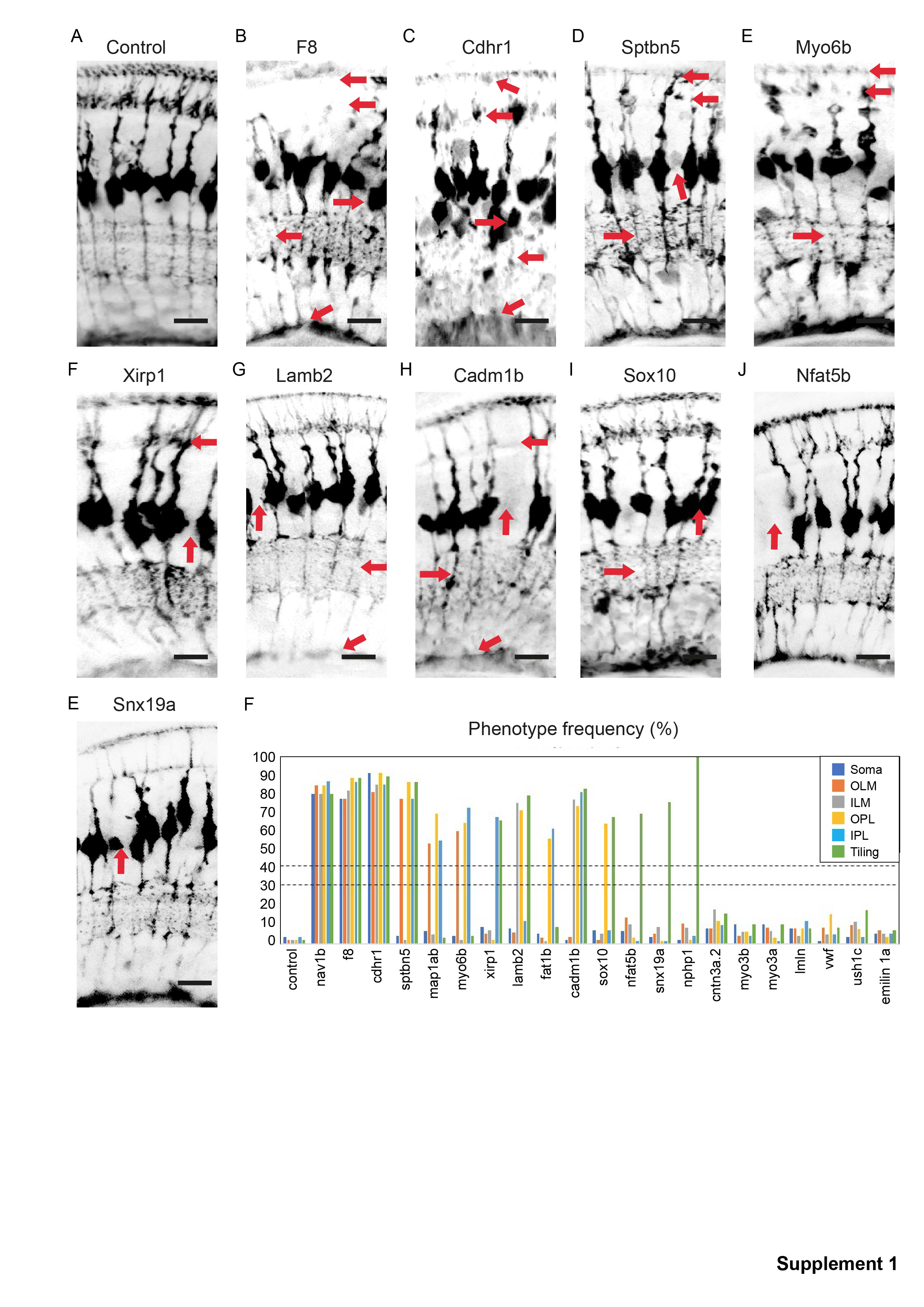

Supplement: Supplementary file 1 — Supplemental figure 1: Phenotypes of gene mutants enriched over windows of MG cell differentiation. (a) slc45a5 controls have no observable MG phenotype. (b) f8 mutants have defects in cell body position, OLM, ILM, IPL, OPL, and tiling. (c) cdhr1 mutants have defects in cell body position, OLM, ILM, IPL, OPL, and tiling. (d) sptbn mutants have defects in OLM, IPL, OPL, and tiling. (e) mapa1b mutants have defects in OLM, IPL, and OPL. (f) xirp1 mutants have defects in OPL and tiling. (g) lamb2 mutants have defects in ILM, IPL, and tiling. (h) Cadm1b mutants have defects in ILM, IPL, OPL, and tiling. (i) sox10 mutants have defects in IPL and tiling. (j) nfat5 mutants have tiling defects. (k) snx19a mutants have tiling defects. (l) Percentages of individual phenotypes observed in all animals from this screen. Dashed lines represent levels of significance from Fisher's exact test after Boniforni multiple test correction (bottom = p < 0.05, top = p < 0.001). Scale bars = 8μm. [file GLIA-67-1401-s001.tif]

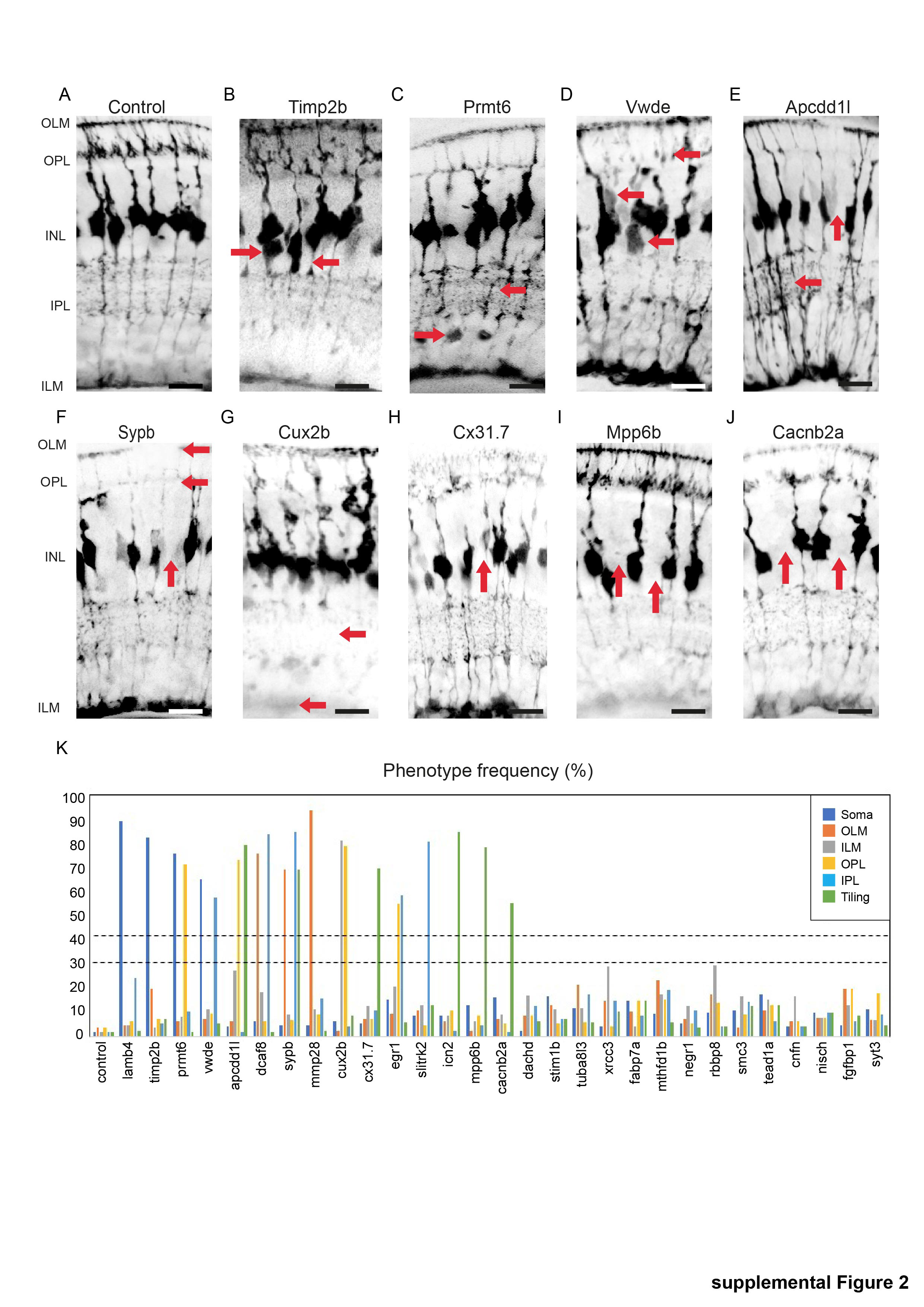

Supplement: Supplementary file 2 — Supplemental figure 2: Phenotypes of gene mutants that are enriched at specific times of MG differentiation. (a) slc45a5 controls have no observable MG phenotype. (b) timp2b mutants have defects in cell body position. (c) prmt6 mutants have defects in cell body position and ILM. (d) vwde mutants have defects in cell body position, OLM, ILM, IPL, OPL, and tiling. (e) apcdd1l mutants have defects in IPL and OPL. (f) sypb mutants have defects in OLM, OPL, and tiling. G) Cux2b mutants have defects in ILM and IPL. (h) cx31.7 mutants have defects in tiling. (i) Mpp6b mutants have defects in tiling. (j) cacnb2a mutants have defects in tiling. (h) Percentages of individual phenotypes observed in all animals from this screen. Dashed lines represent levels of significance from Fisher's exact test after Boniforni multiple test correction (bottom = p < 0.05, top = p < 0.001). Scale bars = 8μm. [file GLIA-67-1401-s002.tif]

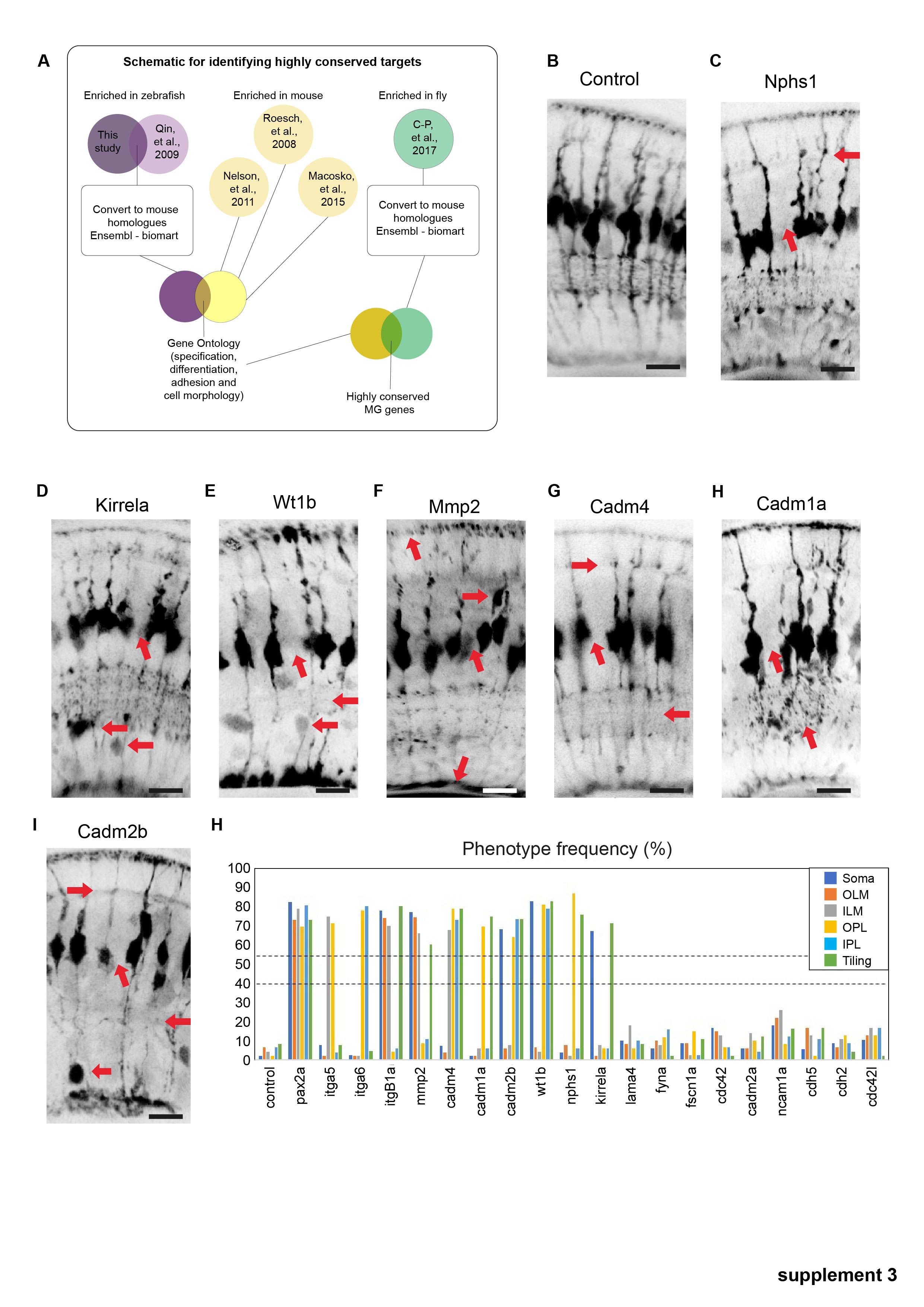

Supplement: Supplementary file 3 — Supplemental figure 3: Phenotypes of conserved highly conserved MG cell genes. (a) Schematic representation of how highly conserved genes we bioinformatically identified. (b) slc45a5 controls have no observable MG phenotype. (b) timp2b mutants have defects in cell body position. (c) nphs1 mutants have defects in ILM, IPL, and tiling. (d) kirrela mutants have defects in cell body position and tiling. (e) wt1 mutants have defects in cell body position, IPL, OPL, and tiling. (f) mmp2 mutants have defects in OLM, ILM, and tiling. (g) cadm4 mutants have defects in OPL, IPL, ILM, and tiling. (h) Cadm1a mutants have defects in IPL and tiling. (i) cadm2b mutants have defects in a cell body positing, IPL OPL and tiling. (j–l) Percentages of individual phenotypes observed in all animals from this screen. Dashed lines represent levels of significance from Fisher's exact test after Boniforni multiple test correction (bottom = p < 0.05, top = p < 0.001). Scale bars = 8μm. [file GLIA-67-1401-s003.tif]

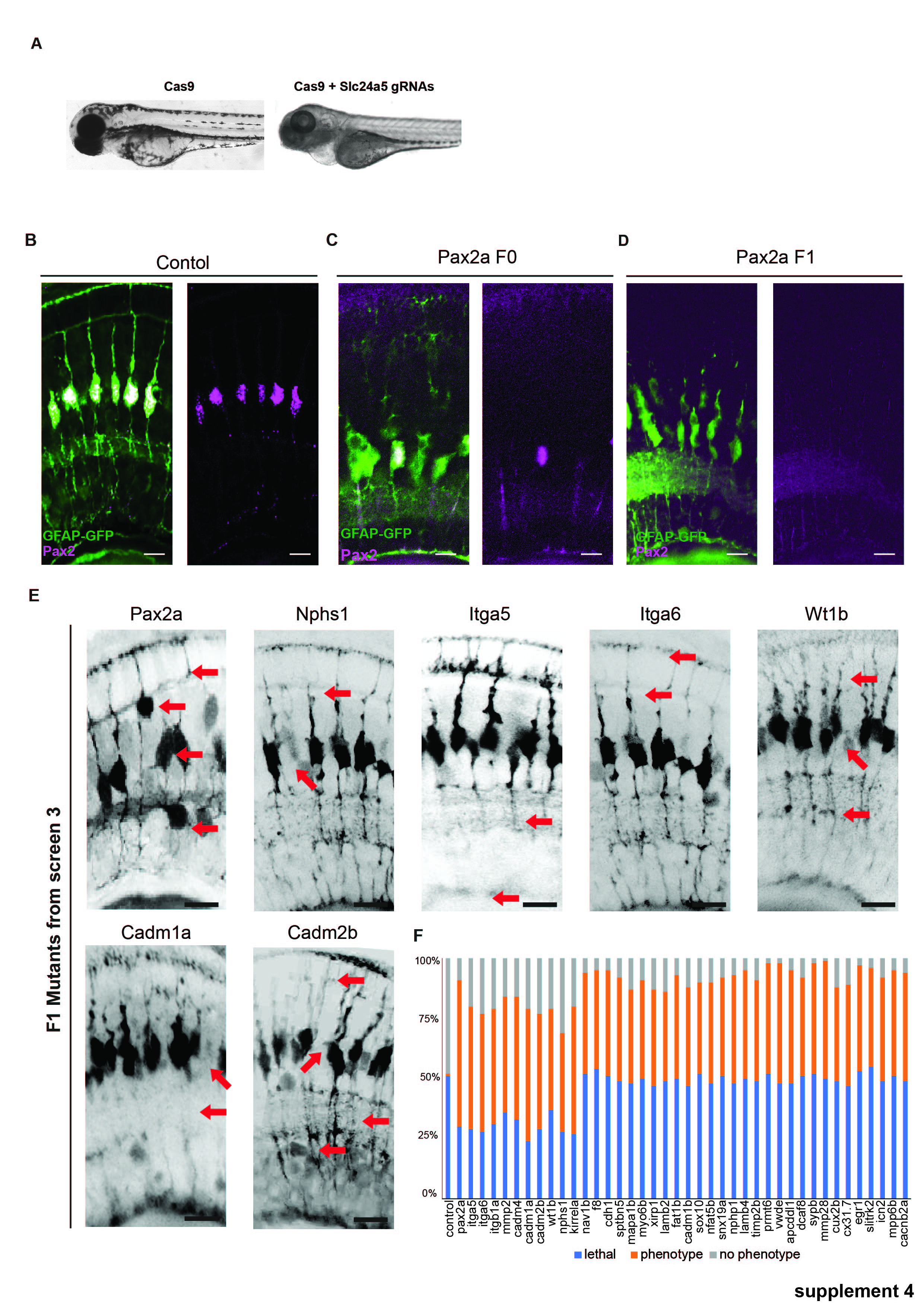

Supplement: Supplementary file 4 — Supplemental figure 4 CRISPR injection validation. (a) Cas9 only injected F0 fish have normal pigmentation at 120hpf while those injected with Cas9 and the slc45a5 guide RNAs are mostly devoid of pigment. (b) In control animals (GFAP:GFP) Pax2 is expressed in all MG by 120hpf. (c) F0 pax2a CRISPR injected animals lack Pax2 expression in most, but not all MG. (d) F1 pax2a CRISPR injected animals Pax2 is absent from all MG. (e) F1 CRISPR mutants with confirmed mutations have notably similar defects to those identified in F0 screen 3. (f) Percentages of animals with lethality and phenotypes after injections. Scale bars = 8μm. [file GLIA-67-1401-s004.tif]
